# Clinical and molecular findings in a Moroccan family with Jervell and Lange-Nielsen syndrome: a case report

**DOI:** 10.1186/s13256-017-1243-1

**Published:** 2017-04-02

**Authors:** N. Adadi, N. Lahrouchi, R. Bouhouch, I. Fellat, R. Amri, M. Alders, A. Sefiani, C. Bezzina, I. Ratbi

**Affiliations:** 1 Centre de Génomique Humaine, Faculté de Médecine et Pharmacie, Mohammed V University, Rabat, Morocco; 2 Département de Génétique Médicale, Institut National d’Hygiène, Rabat, Morocco; 3 Department of Clinical and Experimental Cardiology, Academic Medical Center, University of Amsterdam, Amsterdam, Netherlands; 4 Electrophysiologie et Stimulation Cardiaque, Clinique Belvédère, Rabat, Morocco; 5 Service de Cardiologie B, CHU Ibn Sina, Rabat, Morocco; 6 Department of Clinical Genetics, Academic Medical Center, University of Amsterdam, Amsterdam, Netherlands

**Keywords:** Jervell and Lange-Nielsen syndrome, Long QT syndrome, Deafness, Moroccan, Mutation

## Abstract

**Background:**

Jervell and Lange-Nielsen syndrome (Online Mendelian Inheritance in Man 220400) is a rare autosomal recessive cardioauditory ion channel disorder that affects 1/200,000 to 1/1,000,000 children. It is characterized by congenital profound bilateral sensorineural hearing loss, a long QT interval, ventricular tachyarrhythmias, and episodes of *torsade de pointes* on an electrocardiogram. Cardiac symptoms arise mostly in early childhood and consist of syncopal episodes during periods of stress, exercise, or fright and are associated with a high risk of sudden cardiac death. Jervell and Lange-Nielsen syndrome is caused by homozygous or compound heterozygous mutations in *KCNQ1* on 11p15.5 or *KCNE1* on 1q22.1-q22.2.

**Case presentation:**

We report the case of a 10-year-old Moroccan boy with congenital hearing loss and severely prolonged QT interval who presented with multiple episodes of syncope. His parents are first-degree cousins. We performed Sanger sequencing and identified a homozygous variant in *KCNQ1* (c.1343dupC, p.Glu449Argfs*14).

**Conclusions:**

The identification of the genetic substrate in this patient confirmed the clinical diagnosis of Jervell and Lange-Nielsen syndrome and allowed us to provide him with appropriate management and genetic counseling to his family. In addition, this finding contributes to our understanding of genetic disease in the Moroccan population.

## Background

Jervell and Lange-Nielsen syndrome (JLNS), Mendelian Inheritance in Man (MIM) 220400, is a rare autosomal recessive cardioauditory ion channel disorder that affects 1/200,000 to 1/1,000,000 children [1, 2]. It is characterized by congenital profound bilateral sensorineural hearing loss (SNHL), a long QT interval usually greater than 500 ms, ventricular tachyarrhythmias, and episodes of *torsade de pointes* on an electrocardiogram (ECG) [2, 3]. Cardiac symptoms mostly arise in early childhood and consist of syncopal episodes during periods of stress, exercise, or fright with a high risk of sudden cardiac death [2]. Homozygous or compound heterozygous loss-of-function mutations in *KCNQ1* on 11p15.5 are responsible for 90% of cases of JLNS [4, 5]. Biallelic mutations in *KCNE1* on 1q22.1-q22.2 have been identified as an additional cause of JLNS, establishing the genetic heterogeneity of the disease [6]. The two genes encode respectively the α subunits and β subunits of the voltage-gated potassium channel that in the heart conducts the slow delayed rectifying potassium ion (K^+^) current during cardiomyocyte repolarization, while in the ear it is involved in potassium-rich endolymph production of inner ear hair cells [5, 7]. Here we report the clinical and molecular analysis of a Moroccan family affected by JLNS.

## Case presentation

**Fig. 1:**
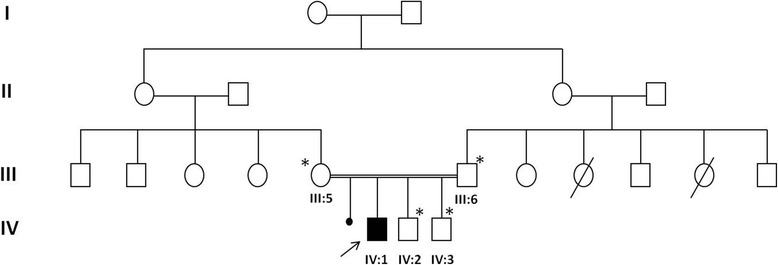
Pedigree of the studied family. The affected individual is shaded and indicated by an *arrow*. Family members that were tested for the mutation are marked by an *asterisk*

**Fig. 2:**
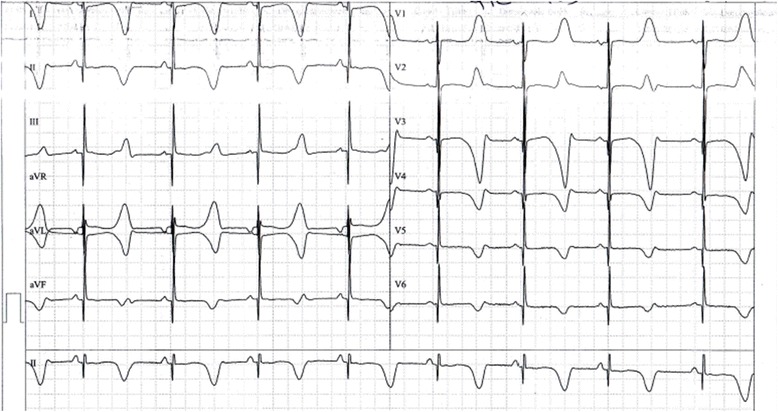
Electrocardiogram of the patient

**Fig. 3:**
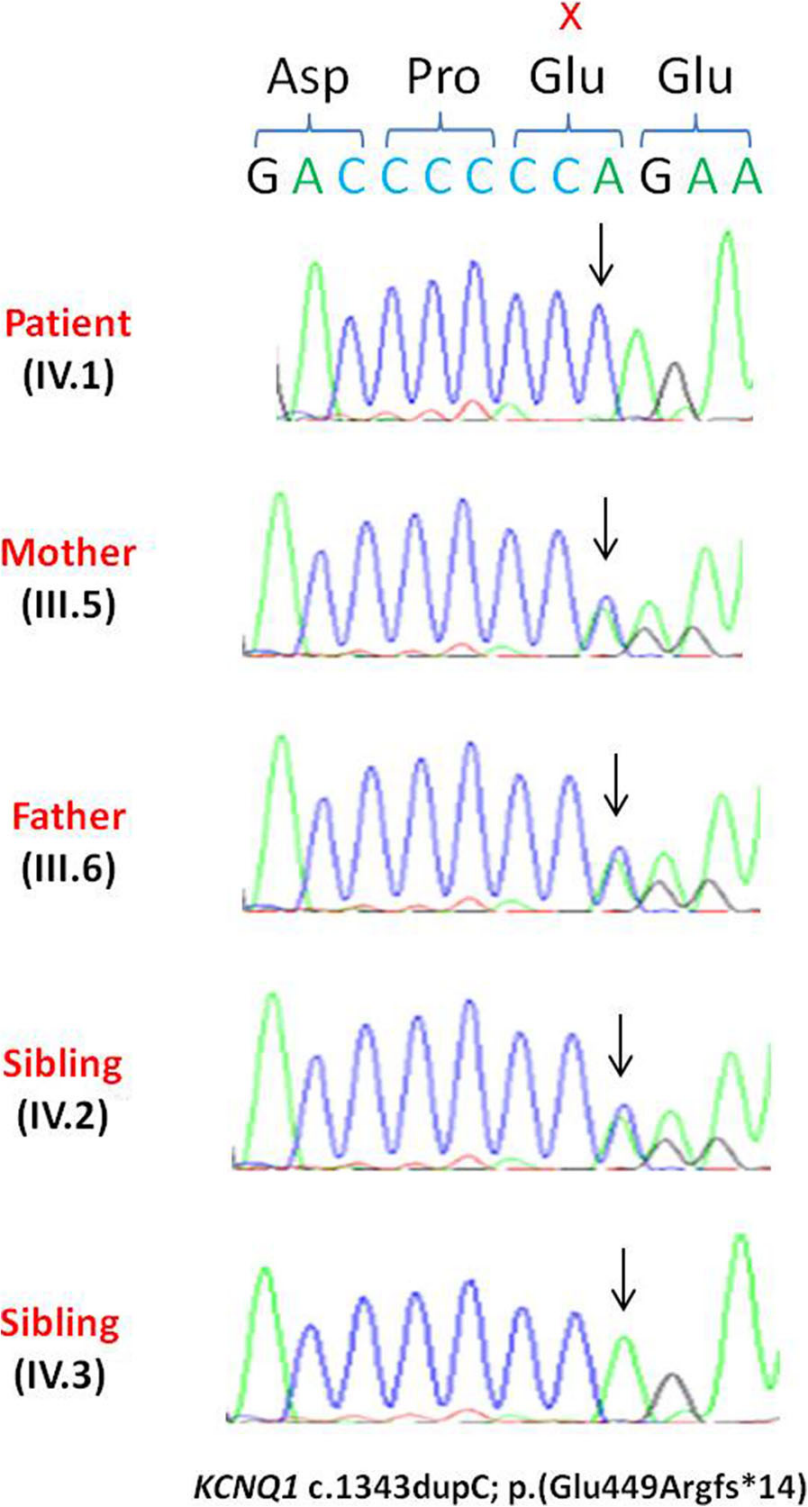
Electropherograms of the identified c.1343dupC; p.Glu449Argfs*14 mutation. The proband IV:1 presented with the homozygous c.1343dupC; p.Glu449Argfs*14 mutation and both parents (III:5 and III:6) and unaffected brother IV:2 are heterozygotes. One healthy brother (IV:3) was homozygous for the wild-type allele. The *X* indicates the position of detected mutation (Duplication of C base). The *arrows* indicate the location of the mutated base

A 10-year-old Moroccan boy was referred by his cardiologist to our medical genetics department (Institut National d’Hygiène, Rabat) for genetic evaluation. He is the firstborn of a healthy consanguineous couple (first-cousins; Fig. 1), both originating from the Northwest of Morocco. There was no family history of sudden death, deafness, syncope, epilepsy, or any other genetic disease. The pregnancy had been medically followed, and no complications were reported. His mother presented with no history of drug ingestion or phytotherapy. His birth weight and length were within normal range and no dysmorphic signs were recorded. At 6 months, he was diagnosed as having severe bilateral SNHL on auditory evoked potential measurement. His first syncopal episode occurred at 24 months of age. His ECG revealed a markedly prolonged QTc interval of 530 ms (corrected by Bazett’s formula) and T-wave alternans on V1 to V4 (Fig. 2). Echocardiography showed a structurally normal heart. Treatment was immediately started with a β-adrenergic blocker. His parents and his two younger brothers, who were 7-years old and 1-year old, were clinically normal. Blood samples from all his family’s members were collected after we were given written informed consent. Deoxyribonucleic acid (DNA) was isolated using standard techniques [8]. Molecular genetic testing of the entire coding region and flanking intronic regions of *KCNQ1* and *KCNE1* was undertaken by Sanger sequence analysis (details available on request). This led to the identification of a homozygous frameshift mutation c.1343dupC (p.Glu449Argfs*14) in the index patient. Both parents and one sibling (IV-2) were heterozygous for this mutation. The youngest child of the family did not carry the frameshift mutation (IV-3, Fig. 3). This variant was previously reported in a heterozygous state in an individual with long QT syndrome (LQTS) [9].

## Discussion

The primary electric disorders, which among others include LQTS, short-QT syndrome (SQTS), Brugada syndrome (BrS), and catecholaminergic polymorphic ventricular tachycardia (CPVT), are often characterized by specific ECG abnormalities either at baseline or during particular conditions, such as exercise (for example, CPVT and LQTS), fever (for example, BrS), or pharmacological challenge (for example, BrS). The list of familial arrhythmia syndromes has been in recent years expanded by the recognition of two other disorders, namely early repolarization syndrome and idiopathic ventricular fibrillation (VF) [7].

LQTS is a clinically and genetically heterogeneous disorder. Syncopal episodes may occur from infancy through middle age, with risk of sudden death [10]. The autosomal dominant mode of inheritance is typical for all LQTS forms (previously described as the Romano–Ward syndrome). Three genes account for approximately 90% of patients with genotype-positive LQTS. LQT1 is characterized by broad-based T waves and cardiac events during exercise or emotion. It arises from loss-of-function mutations in *KCNQ1.* In LQT2, T waves are bifid and cardiac events predominantly occur during exercise or emotion. It arises from loss-of-function mutations in *KCNH2* (also known as *hERG*). Gain-of-function mutations in *SCN5A* are associated to LQT3. These patients show a long ST segment, short T waves, and experience cardiac events predominantly during rest or sleep. Twelve additional genes encoding either ion channel subunits (*KCNJ5*, *KCNE1*, *KCNE2*, and *SCN4B*) or proteins that regulate ion channel function (*AKAP9*, *CAV3*, *ANKB*, *SNT1*, *CALM1*, and *CALM2*) have been associated with LQTS; however, most of them are only rarely implicated (<1%) [7].

Jervell and Lange-Nielsen syndrome is a rare clinical variant of LQTS that manifests with extracardiac phenotypes and is inherited in an autosomal recessive fashion. Patients with JLNS present with severe prolongation of the QT interval and congenital SNHL. It is one of the most severe forms of LQTS. By the age of 3 years, 50% of patients have had an event and by the age of 18 years, 90% of patients with JLNS have developed symptoms [2]. Therefore, the real incidence of JLNS is probably underestimated because of its high mortality in early infancy, in particular in populations with a high rate of consanguineous marriages like Morocco [11]. In addition, even in the presence of medical therapy the occurrence of sudden cardiac death in JLNS exceeds 25% [2].

**Fig. 4:**
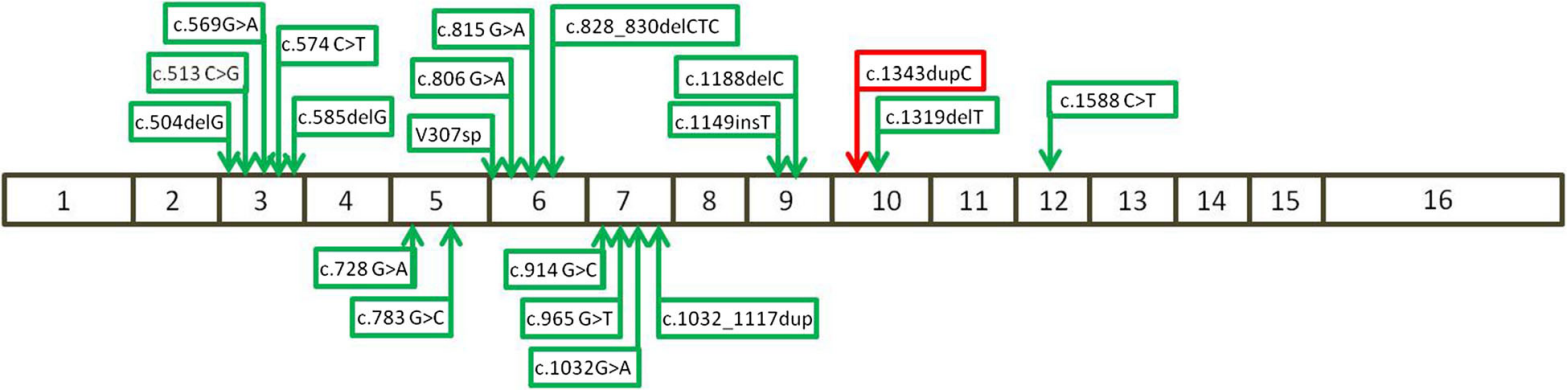
Schematic view of *KCNQ1* gene structure and localization of identified *KCNQ1* mutations in Jervell and Lange-Nielsen syndrome. The mutations previously reported are indicated with a *green frame* and the mutation identified in this study is indicated with a *red frame*

To date, approximately 21 distinct *KCNQ1* mutations have been characterized in patients with JLNS according to The Human Gene Mutation Database (HGMD) at the Institute of Medical Genetics in Cardiff (HGMD, http://www.hgmd.cf.ac.uk/ac/index.php). Three different mutations in the *KCNE1* gene were also reported in patients with JLNS. These mutations have been found homozygously or in a compound heterozygous state (Fig. 4) and, interestingly, one of the families studied in the initial description of *KCNE1* in JLNS originated from Morocco [4].

In this study, we reported the molecular characterization of a *KCNQ1 homozygous* frameshift mutation c.1343dupC (p.Glu449Argfs*14) in a Moroccan patient with JLNS.

This variant was previously identified in a heterozygous state in a 25-month-old girl of Latino origin with severe bilateral SNHL due to a homozygous mutation of connexin 26. She was repeatedly found to have QTc intervals ≥450 ms in a screening program. Sequencing of 12 LQTS genes identified a *de novo* heterozygous frameshift mutation described in this report (*KCNQ1*, c.1343dupC; p.Glu449Argfs*14) [9]. To the best of our knowledge, the case of the Moroccan proband reported here is the first case of JLNS carrying this mutation in a homozygous state.

## Conclusions

We report here the clinical and molecular description of a Moroccan patient with JLNS. This diagnosis allowed us to provide an appropriate course of management to the patient and to identify and counsel asymptomatic heterozygous carriers.
